# Sperm Proteome Maturation in the Mouse Epididymis

**DOI:** 10.1371/journal.pone.0140650

**Published:** 2015-11-10

**Authors:** Sheri Skerget, Matthew A. Rosenow, Konstantinos Petritis, Timothy L. Karr

**Affiliations:** 1 Center for Infectious Diseases and Vaccinology, The Biodesign Institute, Arizona State University, Tempe, Arizona, United States of America; 2 Center for Proteomics, Translational Genomics Research Institute, Phoenix, Arizona, United States of America; Ottawa Hospital Research Institute and University of Ottawa, CANADA

## Abstract

In mammals, transit through the epididymis, which involves the acquisition, loss and modification of proteins, is required to confer motility and fertilization competency to sperm. The overall dynamics of maturation is poorly understood, and a systems level understanding of the complex maturation process will provide valuable new information about changes occurring during epididymal transport. We report the proteomes of sperm collected from the caput, corpus and cauda segments of the mouse epididymis, identifying 1536, 1720 and 1234 proteins respectively. This study identified 765 proteins that are present in sperm obtained from all three segments. We identified 1766 proteins that are potentially added (732) or removed (1034) from sperm during epididymal transit. Phenotypic analyses of the caput, corpus and cauda sperm proteomes identified 60 proteins that have known sperm phenotypes when mutated, or absent from sperm. Our analysis indicates that as much as one-third of proteins with known sperm phenotypes are added to sperm during epididymal transit. GO analyses revealed that cauda sperm are enriched for specific functions including sperm-egg recognition and motility, consistent with the observation that sperm acquire motility and fertilization competency during transit through the epididymis. In addition, GO analyses revealed that the immunity protein profile of sperm changes during sperm maturation. Finally, we identified components of the 26S proteasome, the immunoproteasome, and a proteasome activator in mature sperm.

## Introduction

Advances in mass spectrometry and bioinformatics have greatly increased our understanding of sperm composition and function. Sperm proteome data now exists for several mammalian species, including the mouse, rat, human, macaque and bull [1–7]. While a better understanding of the composition of mature sperm is emerging, our understanding of the complex post-testicular sperm maturation process in mammals is considerably lacking. In this study, we use proteomics to inform a systems-level understanding of the complex maturation process that occurs in the mammalian epididymis.

In mammals, sperm mature and gain fertilization competency as they traverse a specialized duct called the epididymis. This tissue can be generally separated into three distinct but conserved morphological segments termed the caput (proximal), corpus (middle), and cauda (distal) epididymis. When sperm leave the testis and enter the caput epididymis, they are considered immature and are incapable of fertilization. During epididymal transit, sperm lose or modify a number of their surface proteins and gain additional transient or permanent surface proteins in a well-organized manner. To date, a small number of proteins including CRISP1, ADAM7, GPX5 and SPAM1 have been identified as added to sperm during epididymal transit [8–11].

Although it is well accepted that modification of sperm during epididymal transit ultimately confers both motility and fertilization competency to sperm, the process remains poorly understood [12]. One mechanism by which sperm are modified during epididmal transit is by membranous vesicles called epididymosomes which are secreted by the epididymal epithelium (reviewed in [13]). Epididymosomes collected from the epididymal lumen have been shown to contain many proteins that have also been identified as components of sperm. Epididymosomes are thought to interact with sperm during epididymal transit and play a role in transferring proteins to sperm during epididymal transit [14]. The epididymis also contains a number of distinct microenvironments that interact with sperm, although a detailed understanding of the role of these microenvironments remains to be elucidated (reviewed in [15]).

Expressional profiling and principal component analysis identified 6 transcriptionally distinct segments in the mouse epididymis [16]. However, because microarray data may not necessarily directly reflect protein levels (reviewed in [17]), it is important to correlate transcriptomic data with concomitant changes in sperm composition to provide a thorough understanding of the sperm maturation process. An overall understanding of the process would therefore benefit from a detailed analysis of these segmental sperm proteomes.

A number of MS studies have previously characterized the mouseSP at different stages of development and in various subcellular compartments, including the cell membrane, acrosome, and accessory structure of the flagellum [18,19]. A recent study identified 2116 proteins in haploid germ cells undergoing spermiogenesis [20]. A previous study of the mouse caputSP identified 205 proteins [4]. Several studies have also characterized the proteome of sperm isolated from the cauda epididymis, identifying between 858 and, more recently, 2850 proteins [2,21].

In this study, we employed high-throughput MS/MS to characterize changes in the mouse sperm proteome (mouseSP) in the caput, corpus and cauda epididymis. This approach identified 1536, 1720 and 1234 proteins in each segment, respectively. Overall, 2221 different proteins were identified across all three segments. Of the 2221 proteins, only 765 sperm proteins were common to all three segments, suggesting that a substantial number of proteins were either added (732 proteins) or removed (1034 proteins) during epididymal transit. To our knowledge, this is the first study to investigate the corpusSP and to identify the SP from multiple segments of the epididymis in the same study. Combined with associated network and bioinformatic analyses, these results provide a general, qualitative view of the timing, assembly and remodeling of the sperm during maturation.

Gene Ontology (GO) analyses revealed that proteins associated with immune processes change during sperm maturation. Immunity proteins identified in cauda sperm, including complement regulatory proteins CD46, CD55, and CD59b, may play a role in modulation of immune responses in the female reproductive tract. Our analyses also identified components of the 26S proteasome, immunoproteasome, and an alternate proteasome activator in mature sperm which raises the possibility that multiple proteasome types are present in mature sperm.

## Materials and Methods

### Tissue preparation and isolation of sperm

Intact epididymides were harvested from five euthanized adult male mice (*Mus musculus*) and placed in PBS on ice. Each epididymis was then defatted, separated from the testis and vas deferens and cut into three segments, corresponding to the caput, corpus, and cauda epididymis, based on morphological analysis. Tissue samples were pooled, resulting in three tubes each containing 10 caput, corpus or cauda segments in phosphate-buffered saline (PBS). Tissue from each segment was gently macerated using a hand homogenizer and left on a rocker on ice for 30 minutes. The supernatant was drawn off, from which sperm were purified using repeated round of centrifugation and pellet resuspension. Following each pellet resuspension, sperm purity was assessed using a DNA-based fluorescence assay. Equal 5μl aliquots of sperm were mounted on a microscope slide along with an equal volume of PBS containing 1.0μg/ml of 4',6-diamidino-2-phenylindole (DAPI; Invitrogen) and subsequently examined using fluorescence microscopy. Use of animals in this study was approved by the Institutional Animal Care and Use Committee (IACUC) at Arizona State University. All animal procedures followed Protocol Number 11-1168R issued by the ASU IACUC Committee dated 12/17/2010.

### Protein Solubilzation and Quantitation

Sperm samples were solubilized in SDS and quantified using the EZQ^®^ Protein Quantitation Kit (Invitrogen, Inc). Protein fluorescence was measured using a Typhoon Trio+ (Amersham Biosciences/GE Healthcare) equipped with a 488nm laser and 610nm bandpass filter. ImageQuant^™^ TL software was used to analyze fluorescence data. A standard curve was generated using fluorescence data from control samples of known concentration and used to determine sperm sample concentration.

### 1-Dimensional SDS-PAGE

A 1mm 10% NuPAGE^®^ Novex^®^ Bis-Tris Mini Gel was set up using the XCell SureLock Mini-Cell system (Invitrogen) as per manufacturer’s instructions for reduced samples. 50μg of mouse sperm from each of the caput, corpus, and cauda epididymis were loaded and the gel was run for 35 minutes at a 200V constant. Following electrophoresis, the gel was stained using SimplyBlue^™^ SafeStain (Invitrogen) and destained as per manufacturer’s instructions. The gel was transferred to a gel slicer (built in house) and each of the 3 lanes was separated from the rest of the gel by cutting vertically. Each lane was then cut horizontally into 16 equal gel slices. Each gel slice was further cut into smaller (approximately 1x1x1mm) pieces which were transferred to a 0.6mL microcentrifuge tube (16 tubes per segment, for 48 total tubes) and were stored at -80°C until needed.

#### In-Gel Digestion of Proteins / MS Analysis

A standard in-gel digestion protocol was performed on each gel slice as described previously [22]. Gel slices were further destained using 50mM ammonium bicarbonate/50% acetonitrile and dehydrated with 100% acetonitrile. Proteins were reduced and alkylated by treatment with dithiothreitol and iodoacetamide and subsequently digested using 20ng/μl trypsin. Protein digests were extracted with 5% formic acid and 50% acetonitrile, and dried down using a vacuum centrifuge. Resulting peptides were reconstituted in 20μl of 0.1% formic acid.

Extracted peptides were analyzed by nanoflow reverse phase liquid chromatography using a nanoAcquity LC system (Waters) coupled in-line with the linear trap quadrupole (LTQ) Orbitrap Velos instrument (Thermo Fisher Scientific). The nano LC system included a Symmetry C18 5μm 180μm×20mm trap column and a BEH130 C18 1.7μm 100μm×100mm analytical column (Waters). Mobile phases A and B consisted of 0.1% formic acid in water and 0.1% formic acid in acetonitrile, respectively. Samples were loaded onto the trap column for desalting and pre-concentration with 99%:1% mobile phase A:B at a flow rate of 5μl/min for 3 minutes. Peptides were separated on the analytical column at a flow rate of 500nl/min with a two-step linear gradient consisting of 7% B to 25% B in 72 minutes and 25% B to 45% B in 10 minutes. The electrospray ion source consisted of a nanospray head (Thermo Fisher Scientific) coupled with a coated PicoTip fused silica spray tip with OD 360μm, ID 20μm, and 10μm diameter emitter orifice (New Objective, Inc.). Samples were analyzed using positive ion spray voltage and heated capillary temperature of 1.9kV and 220°C, respectively.

Mass spectrometry data were collected with the instrument operating in data dependent MS/MS mode. MS survey scans (m/z 300–2000) were acquired in the Orbitrap analyzer with a resolution of 60,000 at m/z 400 and an accumulation target of 1×10^6. This was followed by the collection of MS/MS scans of the 15 most intense precursor ions with a charge state ≥2 and an intensity threshold above 500 in the LTQ with the accumulation target of 10,000, an isolation window of 2Da, normalized collision energy of setting of 35%, and activation time of 30ms. The tandem MS spectrum was acquired with dynamic exclusion using a repeat count of 1, within a 30 second repeat duration period, and exclusion duration period of 60 seconds.

#### Peptide identification and protein annotation

The mass spectra data files were analyzed using X!Tandem (The GPM; version 2009.10.01.1) searched against the NCBI *Mus musculus* protein fasta file (dated 3/4/2011, with 29617 entries). X!Tandem was searched with a fragment ion mass tolerance of 0.50Da and a parent ion tolerance of 2.0Da. Iodoacetamide derivative of cysteine was specified as a fixed modification, while oxidation of methionine was specified as a variable modification. Results were merged using Scaffold (Proteome Software) version 3.4.9 in which protein probability False Discovery Rates (FDRs, <1%) were calculated by using the assigned protein probabilities estimated from the results of Peptide and Protein Prophet. Peptide identifications were accepted if they could be established at greater than 90.0% probability as specified by the Peptide Prophet algorithm [23] and protein identifications were accepted if they could be established at greater than 99.0% probability and contained at least 2 identified peptides. Protein probabilities were assigned using the Protein Prophet algorithm [24]. Proteins that contained similar peptides and could not be differentiated based on MS/MS analysis alone were grouped to satisfy the principles of parsimony.

Protein identifications from Scaffold after analysis with X!Tandem consisted of Mouse RefSeq (NCBI) protein IDs. These protein IDs were entered into BioMart (biomart.org, version 0.7) using the Ensembl Genes 69 (Sanger UK) *Mus musculus* genes (GRCm38) to obtain cross-referenced Ensembl Gene IDs and associated gene names.

### Phenotype Analysis

Ensembl gene IDs for proteins identified in the caputSP, corpusSP and caudaSPs were inputted into Biomart using the Ensembl Genes 69, Sanger UK database to obtain corresponding MGI IDs. The resulting MGI IDs were inputted into Biomart using the MGI, Jackson Laboratory US, Genes & Genome Features database to obtain corresponding phenotype IDs and phenotype terms. These results were curated to include phenotype terms associated with abnormal sperm morphology or processes and tabulated.

#### Gene Ontology Analysis

Gene group functional profiling (g:GOSt) was used to individually analyze the Gene Ontology (GO) category distribution for the caputSP, corpusSP and caudaSP [25] while the compact compare of annotations (g:Cocoa) function was used to compare the (GO) category distribution across the three datasets [26,27]. For each analysis, the organism was set to *Mus musculus* and Ensembl gene IDs corresponding to the caputSP, corpusSP and caudaSP were used for the query. In total, 1523 caputSP, 1706 corpusSP and 1216 caudaSP Ensembl IDs were input. The significance threshold was set to Benjamini-Hochberg FDR.

#### Network Analysis

All network analyses were conducted using the ClueGo plugin (v2.0.1) for Cytoscape (v3.0.0) [26]. GO Biological Process Annotations (downloaded 19.02.2013) for proteins added (732 proteins) and removed (1034 proteins) were compared using statistical tests for gene enrichment (right-sided hypergeometric test) with Benjamini-Hochberg multiple test correction was implemented. Network parameters were set as follows: GO Tree Levels (min = 1, max = 8), GO term restriction (min#genes = 5, min% = 3), and GO Term Connection Restriction (Kappa score threshold = 0.3). Only terms with a p-value ≤ to 0.05 were shown, and resulting groups consisted of a minimum of 3 terms and groups sharing >50% of terms were merged.

GO Immune System Process Annotations (downloaded 19.02.2013) for proteins found in the caputSP (1536 proteins) and caudaSP (1234 proteins) were compared using statistical tests for gene enrichment (right-sided hypergeometric test) with Benjamini-Hochberg multiple test correction was implemented. Network parameters were set as follows: GO Tree Levels (min = 1, max = 5), GO term restriction (min#genes = 2, min% = 1), and GO Term Connection Restriction (Kappa score threshold = 0.3). Resulting groups consisted of a minimum of 3 terms and groups sharing >50% of terms were merged. It should be noted that curation of the GO Immune System Process Annotations is an ongoing process and thus, some genes, especially species specific immunity genes likely remain unannotated.

The mouse caputSP, corpusSP and caudaSPs contain a number of β-defensins that are not annotated as part of the GO Immune System Process ontology. Since many β-defensins exhibit region specific expression in the epididymis, for each β-defensin identified in this study, we determined the corresponding region of the epididymis with the highest expression based on expression data collected from two previous studies conducted in the mouse and rat [16,28]. Data from Johnston et al. was accessed via the Mammalian Reproductive Genetics website (http://mrg.genetics.washington.edu/). Human orthologs for each of the β-defensins were identified using Ensembl (ensembl.org).

## Results

### Caput, corpus, cauda sperm proteomes

**Fig 1:**
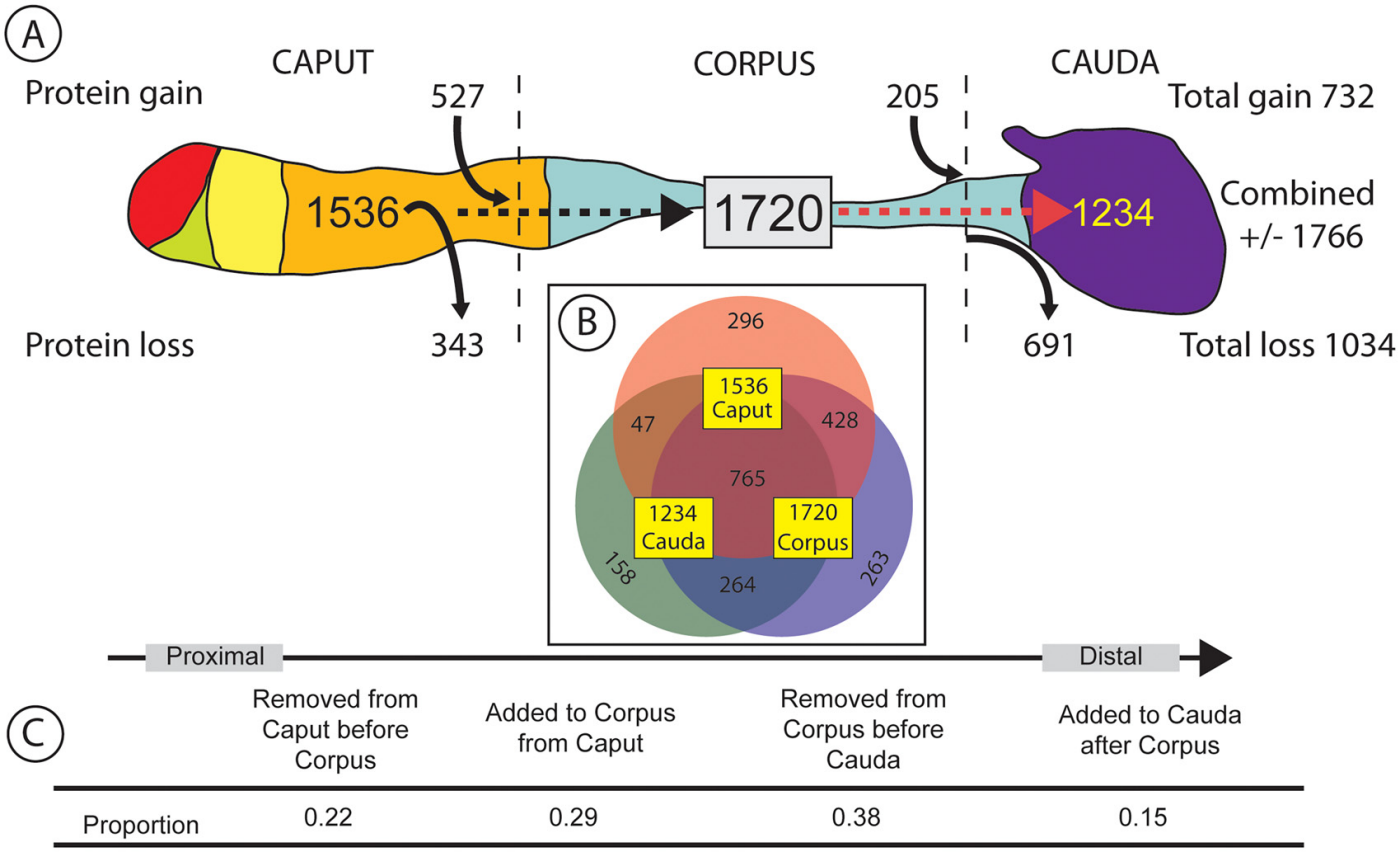
Summary of sperm proteomes derived from the three major regions of the mouse epididymis. (A) Graphical representation of epididymis. Different colors represent the 6 distinct transcriptional segments previously identified [14]. Numbers within the epididymis indicate the number of proteins identified in the caputSP (1536), corpusSP (1720) and caudaSP (1234), respectively. Proteins added (protein gain), or removed (protein loss), from sperm between segments are displayed above and below the epididymis. In total, 732 proteins were added and 1034 proteins were removed (1766 total) from sperm during epididymal transit. (B) Venn diagram illustrating the overlap of protein identification between the three epididymal segments. 765 proteins were found to be common to sperm from all segments. (C) Proportion of proteins gained and lost by segment.

Tryptic peptides from sperm isolated from the caput, corpus and cauda regions of the mouse epididymis were analyzed by mass spectrometry. Our analyses identified 10951, 12634 and 8456 unique peptides and 1536, 1720, and 1234 proteins, respectively, from each of the segments. In total, 2221 different sperm proteins were identified across all three segments with average peptide coverage of ~25 percent/protein (Table 1, S1 Table). The average number of peptide hits per protein across all segments (~7) indicated significant coverage of the major components of the sperm proteome. A provisional ‘core’ sperm proteome of proteins common to all three segments (765 proteins) was also identified by this approach (S1 Table, Fig 1).

**Table 1:** Summary of Mass Spectrometry Results.

	Caput	Corpus	Cauda
Total proteins identified	1536	1720	1234
Total peptides identified	10951	12634	8456
Average percent protein coverage	25.22%	25.19%	25.09%
Average peptide hits per protein	7.13	7.35	6.85

The core sperm proteome was used as a proxy to provisionally identify proteins added to, or removed from, the three regions of the epididymis (Fig 1; S2 Table). This analysis revealed dynamic, global changes in the SP during transit through the epididymis. Compared to the caputSP, the corpusSP contained as many as 527 additional proteins, suggesting these proteins are added to sperm during transit out of the caput. Similarly, the caputSP provisionally contained 343 proteins not detected in the corpusSP, suggesting these proteins are removed during transit. Likewise, the caudaSP contained as many as 486 fewer detectable proteins than the corpusSP (1720 vs. 1234, respectively) the result of 205 proteins not identified in the corpusSP (and therefore assumed to be gained) and 691 corpusSP proteins not identified in the caudaSP (and presumably removed during transit out of the corpus to the cauda epididymis). 47 proteins were identified in the caputSP and caudaSP but not in the corpusSP, representing a small number of proteins that were likely missed by MS analysis of the corpusSP (S2 Table, see Discussion). In summary, these results suggest that the sperm proteome undergoes substantial changes during epididymal transit and overall 732 proteins were acquired and 1034 proteins lost by sperm during epididymal transit.

#### Spectral counts and protein abundance

The 20 most abundant proteins in each of the three segments are ranked in Table 2 by spectral counts, which provide a semi-quantitative estimate of protein abundance [29]. The most abundant class of proteins in the caputSP are keratins (11/20). This list includes LOC239673 that appears, by BLAST searches, to be an unannotated keratin. Although keratins are typically attributed to sample contamination, the majority of these have been found to be present in the human sperm nucleus and were also found to be highly abundant in the Rhesus macaqueSP [7,30]. Keratins not only serve structural roles, but are also thought to play a role in a variety of cellular processes including protein transport, rapid and localized restructuring, differentiation and proliferation (reviewed in [31]). One of the most abundant keratins identified in the sperm proteome, KRT5, is involved in sperm head shaping [32].

**Table 2:** Top 20 proteins in the caputSP, corpusSP and caudaSP by spectral count.

	Caput		Corpus		Cauda	
	Symbol	Count	Symbol	Count	Symbol	Count
1	LOC239673	812.19	AKAP4	1730.39	AKAP4	3391.86
2	KRT5	720.26	ODF2(E)	415.06	LOC100503160	2455.21
3	KRT7	698.63	LOC239673	413.14	ODF1	890.25
4	KRT6A	692.15	ODF2(F)	408.34	ODF2(E)	828.39
5	AKAP4	681.33	KRT73	388.16	ODF2(F)	816.79
6	KRT85	649.97	KRT5	367.99	FABP9	637.97
7	KRT75	647.80	ATP5B	356.46	LDHC	611.87
8	KRT14	617.52	FABP9	355.49	OXCT2B	605.10
9	KRT42	523.44	ATP5A1	343.00	GAPDHS	590.6
10	KRT17	480.18	VDAC2	336.28	AKAP3	572.24
11	ATP5B	456.38	OXCT2B	325.71	TUBB4B	516.17
12	KRT1	445.57	LDHC	317.06	GSTM5	503.61
13	DSP	421.78	KRT6A	315.14	TUBA3A	489.11
14	ATP5A1	389.33	KRT75	308.42	LOC239673	483.31
15	HSPA5	370.95	SLC25A5	306.49	FSIP2	440.78
16	KRT2	367.70	AKAP3	301.69	LDHAL6B	433.04
17	HSP90B1	355.81	KRT14	299.77	TUBA1A	431.11
18	JUP	346.07	FSIP2	287.28	KRT5	419.51
19	Gm4846	336.34	ODF1	281.51	TUBB4A	419.51
20	GOT2	302.81	GOT2	276.71	KRT85	409.85

While keratins are still abundant in the corpusSP (6/20), the most highly abundant proteins are AKAP4 and isoforms of the outer dense fiber protein, ODF2. The most abundant proteins in the caudaSP include AKAP4 and LOC100503160 (AKAP4-like). AKAP4 has consistently been identified as an abundant protein of sperm in a variety of species, and results from this study suggest that AKAP4 increases in relative abundance as sperm traverse the epididymis [1,7]. AKAP-like was only identified in caudaSP, and the very high spectral counts observed for this protein in the cauda strongly suggest this protein is primarily expressed and inserted into the sperm in this distal region of the epididymis. Finally, orthologs of the most abundant cauda sperm proteins (7/20) were similarly identified as the most abundant proteins in sperm obtained from the cauda epididymis of the Rhesus macaque [7].

Comparison of protein content across the three segments revealed that 17/20 of most abundant proteins identified in the caudaSP were also found in the caputSP and corpusSP suggesting that these proteins either increase, or other proteins decrease, in abundance in sperm as they traverse the epididymis. Both possibilities appear to be involved: (i) comparison of the top 20 cauda sperm proteins across all three segments reveals a clear pattern of increases in spectral counts (Table 3; S1 Fig), and (ii) there is a net loss of 691 proteins during transit (Fig 1) resulting in fewer total proteins identified in the caudaSP which could have an additive effect on the observed spectral counts. Based on spectral count comparisons, the top 20 proteins increase approximately 3–7 fold in the caudaSP, suggesting that increased protein expression and insertion into the sperm are the major factors responsible for the increase in this subset of proteins. Thus, as a global indicator of protein abundance and large-scale proteomic changes, categorization by spectral counts appears a valid metric to provide a qualitative and semi-quantitative overall view of the sperm proteome.

**Table 3:** Comparison of top 20 caudaSP proteins (by spectral counts) to the caputSP and corpusSP.

Protein	Counts		
	caput	corpus	cauda
AKAP4	681.33 (5)*	1730.39 (1)	3391.86 (1)
AKAP4-like	-	-	2455.21 (2)
ODF1	122.21 (71)	281.51 (19)	890.25 (3)
ODF2(E)	241.17 (27)	415.06 (2)	890.25 (4)
ODF2(F)	-	408.34 (4)	816.79 (5)
FABP9	114.64 (73)	355.49 (8)	637.97 (6)
LDHC	128.7 (63)	317.06 (12)	611.87 (7)
OXCT2B	121.13 (72)	325.71 (11)	605.1 (8)
GAPDHS	114.64 (74)	273.83 (21)	590.6 (9)
AKAP3	124.45 (66)	301.69 (16)	572.24 (10)
TUBB4B	163.3 (48)	265.18 (24)	516.17 (11)
GSTM5	73.45 (121)	120.1 (68)	503.61 (12)
TUBA3A	143.84 (53)	269.98 (22)	489.11 (13)
LOC239673	812.19 (1)	413.14 (3)	483.31 (14)
FSIP2	78.95 (110)	287.28 (18)	440.78 (15)
LDHAL6B	91.93 (92)	255.57 (26)	433.04 (16)
TUBA1A	134.1 (62)	245.96 (28)	431.11 (17)
KRT5	720.26 (2)	367.99 (6)	419.51 (18)
TUBB4A	135.18 (60)	216.18 (34)	419.51 (19)
KRT85	649.97 (6)	-	409.85 (20)

*Rank of protein for each list is given in parentheses.

#### Sperm Phenotypes

There were 60 sperm proteins identified with known sperm phenotypes including abnormal sperm morphology and abnormal sperm processes (Table 4). One third (20/60) of these proteins were identified in this study as being added to sperm during epididymal transit (Table 4, bold) and include ADAM3, CD46, CD59B, CNPY4, CRISP1, H2-AB1, H2AFX, HSPA4L, MNS1, NPC1, PRKAR1A, RSPH1, SEPT4, SERPINE2, S100A11, SPAM1, SPINK2, THEG, TKT, and TSSK1. A serine protease inhibitor, CRISP1 involved in sperm-egg binding and regulation of capacitation, is known to be secreted into epididymal fluid and has been shown to be associated with cauda, but not caput sperm in the rat [10]. We identified CRISP1 in sperm from both the corpus and cauda epididymis, and previous proteomic studies of identified CRISP1 in caput sperm, suggesting that this protein becomes associated with sperm in the proximal epididymis [4].

**Table 4:** Mouse caput, corpus and cauda sperm proteins with known associated sperm phenotypes.

Phenotype	GO Identifier	Proteins
**Abnormal Morphology**		
Acrosome	2686, 8839, 8898	Ppp1cc, Spaca1, Spesp1, Zpbp
Axoneme	9838	**Mns1** ^a^, Odf2, Spag6, Tekt2, Theg, Vdac3
End piece	9837	Akap4
Flagellum	8545, 8892, 8893, 9237, 9238, 9239, 9242, 9243, 9839	Akap4, Atp1a4, Gpx4, **Mns1**, Npc1, Oaz3, Odf2, Ppp1cc, Prss21, **Sept4**, Spag6, Spaca1, Spink2, Tekt2, Theg, Zpbp
Midpiece	9831	Odf2, Ppp1cc, Spag6
Mitochondrial sheath	9832, 9833	Akap4, Gpx4, **Mns1**, Ppp1cc, **Sept4**, Vdac3
Nucleus	9232	Gpx5, Theg, Zpbp
Principal piece	9836	Akap4, Gpx4, **Mns1**, Ppp1cc, **Sept4**, Spag6
Spermatid	6380	Acox1, Hsd17b4, **Mns1**, Npepps, Ppp1cc, **Rsph1**, Sgpl1, Spaca1, Theg, Tssk1, Zpbp
Sperm head	9230, 9234	**Cd59b**, Fabp9, Gpx4, Npc1, Ppp1cc, Spaca1, Spag6
Sperm physiology	4543	Akap4, **Crisp1**, Ppp1cc, Prss21, **Rsph1**, **Sept4**, **Spam1**, Spesp1, Zan
**Impaired / Abnormal Process**		
Acrosome reaction	4542	**Adam3**, **Npc1**, Mgfe8, Oaz3, Prss21
Capacitation	3666	Atp1a4, **Sept4**
Fertilization	242, 5410, 5411, 9647	**Adam3**, Atp1a4, **Cd46**, Gpx4, **Npc1**, Prss21, **Spam1**, Spesp1
Male fertility / infertility	1921, 1922, 1925, 2161	Acox1, **Adam3**, Akap4, Ascl4, Atp1a4, B4galnt1, Bckdk, Capza3, Cav1,Chdh, Cib1, **Cnpy4**, Dnahc1, Dync1h1, Entpd5, Gatm, Gnpat, Gpx4, Gpx5, **H2-Ab1**, Hsd17b4, **Hspa4l**, Mfge8, **Mns1**, Myo6, Npepps, **Npc1**, Oaz3, Odf2, Pomgnt1, Ppp1cc, **Prkar1a**, Rhcg, Rpl38, **Rsph1**, **Sept4**, **Serpine2**, Sgpl1, Spaca1, Spag6, **Spink2**, Tekt2, **Theg**, **Tkt**, Tpst2, **Tssk1**, Vdac3, Zpbp
Meiosis	4901, 8261	Gnpat, Endpd5, **H2afx**, Ppp1cc
Motility	2674, 2675, 9280, 9282	Akap4, Atp1a4, **Cd59b**, Chdh, Dnahc1, **Hspa4l**, **Mns1**, Npepps, Odf2, Prss21, Sept4, Spaca1, Spag6, Tekt2, **Theg**, **Tssk1**, Vdac3, Zpbp
Spermatogenesis	1155, 1156	Acox1, Cib1, Gatm, Hsd17b4, **Hspa4l**, **Npc1**, Npepps, Prss21, Sgpl1, Spaca1, **Tssk1**, Zpbp
Spermiogenesis	1932, 8279	Cib1, B4galnt1, Ppp1cc, Capza3, **Sept4**
Sperm number	2673, 2687, 5159	Cib1, Gnpat, Capza3, **Hspa4l**, **Mns1**, **Npc1**, Npepps, Oaz3, Ppp1cc, Prss21, **Rsph1**, **S100a11**, **Spink2**, **Tssk1**, Tpst2

^a^Proteins found to be added to sperm during epididymal transit in this study are indicated in **bold**.

### GO Analysis of the Caput, Corpus and Cauda SP

#### Biological Processes

Many biological process GO categories are enriched in the caputSP, corpusSP and caudaSPs that can provide clues about the broad function of different regions of the epididymis. For example, all three are enriched for proteins involved in localization, however the caputSP (p = 7.69 e-45) exhibits a greater enrichment than the corpusSP (p = 2.99 e-30) and caudaSP (p = 1.12 e-10). More specifically, proteins involved in lipid localization are enriched in the caputSP (p = 9.54 e-4) and corpusSP (p = 1.04 e-3), but not in the caudaSP. Proteins involved in protein localization are also enriched in the caputSP (p = 4.33 e-18) and corpusSP (p = 4.09 e-14) but are far less prominent in the caudaSP (p = 2.07 e-3). These results suggest that localization proteins in the caputSP and corpusSP may be involved in sperm remodeling and localization of sperm proteins added during transit through the epididymis.

Proteins involved with translation are enriched in the caputSP (n = 134, p = 1.85 e-32), corpusSP (n = 131, p = 1.52 e-26) and caudaSP (n = 62, p = 5.56 e-5), however the number of proteins identified in this category decrease along the length of the epididymis, especially between the corpus and cauda regions. A recent analysis of the mouse caudaSP also found translation proteins enriched in sperm, suggesting that some level of translation may occur in sperm within the epididymis, or that remnants of translational machinery remains in these cells after testicular maturation [21]. In addition, proteins involved in glycosylation are enriched in the caputSP (n = 35, p = 1.78 e-11) and corpusSP (n = 24, p = 3.78 e-4), but not the caudaSP where only 12 proteins associated with glycosylation are identified. Specifically, we found that proteins involved in glycoprotein biosynthetic processes are only enriched in the caputSP (n = 42, p = 2.65 e-11) and corpusSP (n = 27, p = 5.10 e-3).

#### Cellular Components

A large proportion of proteins identified in the caputSP, corpusSP and caudaSPs are annotated as membrane proteins. Membrane proteins are enriched in the caputSP (n = 859, p = 7.54 e-48), corpusSP (n = 857, p = 2.23 e-27) and caudaSP (n = 571, p = 1.63 e-8), and the number of membrane proteins identified declines as sperm traverse the epididymis, especially from the corpus to cauda. Because many of these proteins are likely found on the sperm surface, this reduction may reflect the pruning of sperm surface proteins during the maturation process.

Proteins associated with the flagellum are also enriched in the caputSP (n = 24, p = 3.61 e-10), corpusSP (n = 32, p = 1.98 e -16) and caudaSP (n = 35, p = 7.21 e-24). This enrichment correlates with an increase in the number of proteins associated with the flagellum along the length of the epididymis. Many of the proteins that appear to be added in the corpus and cauda are sperm specific and have previously been found to play a role in sperm motility. For example, CATSPERB, GAS8, IQUB, SEPT4, Sept12, SORD, TSSK1 and TXNDC8 were identified in corpus, but not caput sperm, while AKAP14, CATSPERG2, CCDC135 and SPA17 were identified in cauda, but not corpus sperm. None of the aforementioned proteins were found in the previously reported mouse caput sperm proteome suggesting that these proteins are acquired by sperm during epididymal transit and that these proteins play a key role in the acquisition of motility [4].

Proteins comprising the proteasome complex are also enriched in the caputSP (p = 8.00 e-1), corpusSP (p = 1.00 e-8) and caudaSP (p = 7.8 e-10). Accordingly, the number of proteasome proteins found in the sperm proteome increases as sperm progress from the proximal to the distal end of the epididymis. In total, 27 proteasome proteins were identified in mouse sperm, including 11 in the caputSP, 23 in the corpusSP, and 23 in the caudaSP. Although the number of proteasome proteins identified in the corpus and cauda are the same, the identified proteins differ and the number in the cauda represents a larger proportion of the overall proteome (23/1345) than in the corpus (23/1817). The 26S proteasome is conventionally involved in the degradation of ubiquitinated peptides and proteins, and is thought to play a role in a variety of processes central to sperm function including sperm remodeling, capacitation, and the acrosome reaction [33,34].

The proteasome proteins identified in this study comprise various components of the 26S proteasome, including the 20S core and 19S regulatory particle, as well as alternate proteasome activators (PA200 & PA28) and proteins which assist in proteasome assembly or regulation. PSMA1-7 and PSMB1-7 comprise 20S core particle, while PSMD1, 2, 6 & 7 are components of the 19S regulatory particle. PSMA8, a sperm-specific paralog of PSMA7, was also identified however its function has yet to be elucidated. PSMG1 & PSMG2 form a heterodimer which promotes assembly of the 20S core subunit and is subsequently degraded by the 20S subunit following assembly [35]. In addition, PSMG1 & 2 likely also plays a role in preventing dimerization of α-rings [36].

PSME4, also known as PA200, is an alternate proteasome activator which has been proposed to replace the 19S regulatory complex at one or both ends of the 20S core particle and play a role in DNA double strand break repair [37,38]. PSME4 exhibits broad tissue expression, with the highest level of expression detected in the testis, and mice lacking PSME4 exhibit marked reduction in male, but not female, fertility as a result of pre- and post-meiotic defects in spermatogenesis, resulting in a reduction of normal sperm [39]. Finally, PSME1 & 2, which comprise the proteasome activator 28 (PA28 complex) were also identified. Like PA200, PA28 binds the 20S core subunit in place of the 19S regulatory particle at one or both ends of the proteasome to form the immunoproteasome complex [40,41]. Identification of PSME1 & 2 (PA28), as well as PSME4 (PA200) in the mouse SP, suggests that both 26S, and alternate proteasome complexes, including immunoproteasomes may exist in sperm.

#### Molecular Functions

Consistent with other sperm proteomes, the majority of proteins in the caputSP, corpusSP and caudaSPs possess binding and/or catalytic functions [4,7,12]. Many binding proteins were identified in the caputSP (n = 943, p = 1.97 e-16), corpusSP (n = 1025, p = 1.44 e-15) and caudaSP (n = 732, p = 5.52 e-08) and largely consist of proteins involved in small molecule binding, cofactor binding, and protein binding. Catalytic proteins are greatly enriched in the caputSP (n = 795, p = 1.15 e-131), corpusSP (n = 812, p = 1.77 e-11) and caudaSP (n = 605, p = 3.91 e-84), and consist largely of proteins with oxidoreductase, hydrolase and transferase activity.

Finally, the caputSP (n = 127, p = 7.7 e-48), corpusSP (n = 131, p = 2.68 e-46) and caudaSP (n = 78, p = 1.90 e-20) are also enriched in proteins that have structural molecule activity. The number of proteins with structural molecule activity is greatly reduced from the corpusSP to caudaSP. It is well known that restructuring and reshaping occurs in sperm as they mature in the epididymis, and thus the reduction in structural molecules in mature sperm may reflect the completion of many of these processes once sperm reach the cauda epididymis.

One particular GO category, “structural component of ribosomes”, is also overrepresented in caputSP (n = 63, 3.31 e-38), corpusSP (n = 69, p = 1.55 e-42) and caudaSP (n = 30, p = 1.10 e-10) sperm. However, the number of proteins identified in the caudaSP greatly decreases, and as discussed above, the number of proteins related to translation also decline substantially from corpus to cauda sperm. This finding supports the idea that some level of translation may occur in sperm, particularly sperm in the caput and corpus, however these processes may be reduced or halted in caudal sperm.

### Added / Removed Proteins

**Fig 2:**
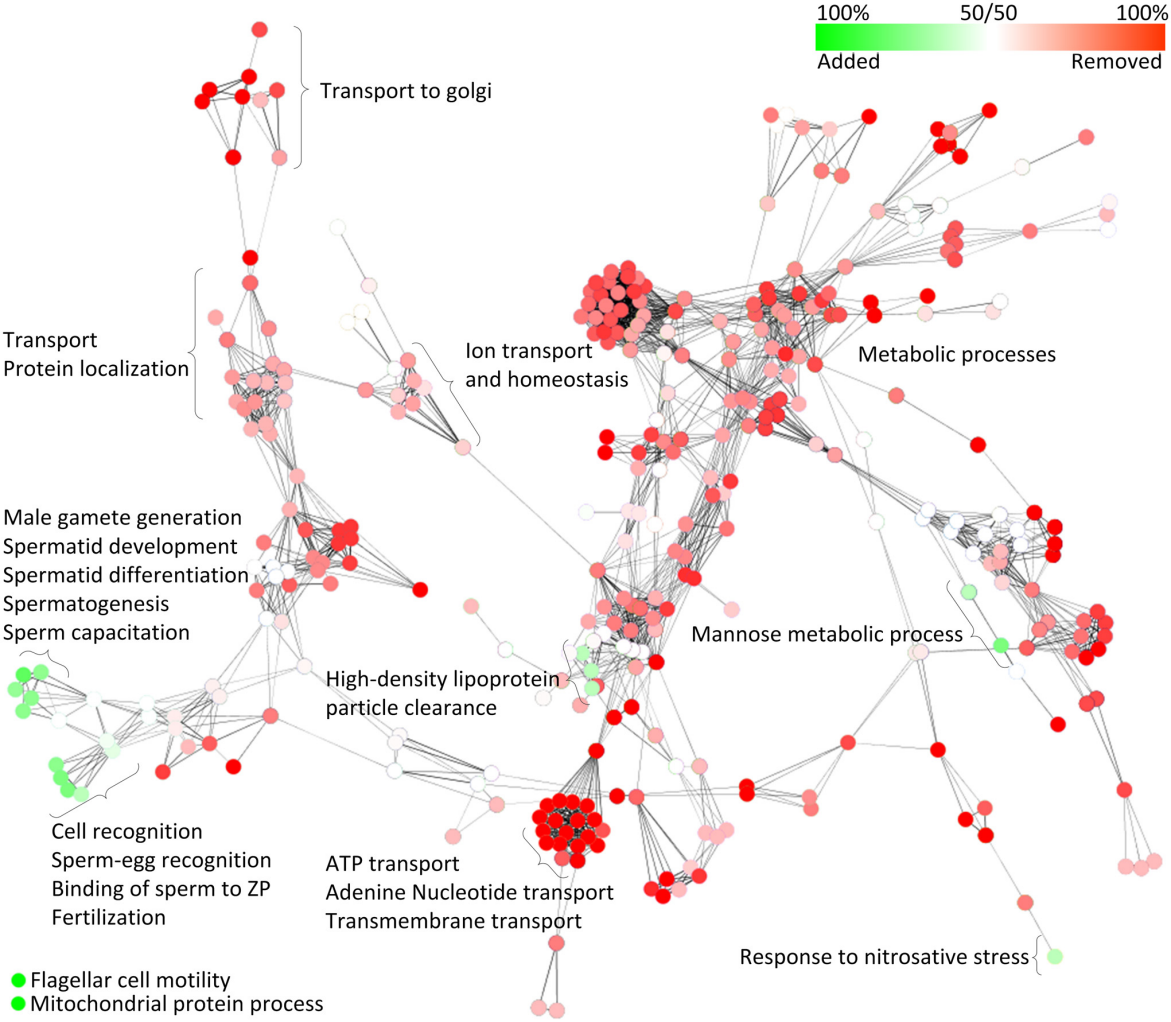
Enriched biological process GO categories for proteins added and removed from sperm during epididymal transit. Network biological process diagram indicating functional enrichment of GO categories for proteins added (green) and removed (red) from sperm during epididymal transit. Color legend at top indicates range of overlap shared by the datasets. The varying shades of color represent nodes and clusters of functional GO categories biased towards proteins added (green) or removed (removed) during transit through the epididymis.

Of the 2221 proteins identified in the caputSP, corpusSP and caudaSP, 765 were found across all three segments (Fig 1). Comparison between proteomes suggest that as many as 732 proteins are added, and 1034 proteins removed, from sperm during transit through the epididymis (total 1766 proteins). In order to further study these 1766 proteins, a comparative network analysis was conducted to find GO Biological Process terms enriched in each list (Fig 2). We found that proteins added to sperm during transit are enriched for several GO categories related to sperm function and development including proteins involved in spermatogenesis, sperm capacitation, sperm-egg recognition, and motility (Table 5). This finding is consistent with the widely accepted role of the epididymis in conferring fertilization competency and motility to sperm. Finally, proteins related to mitochondrial protein processes, high-density lipoprotein particle clearance, mannose metabolic processes and response to nitrosative stress were also found enriched. Proteins removed from sperm during transit are also enriched in a number of GO Biological Process terms including transport to golgi, ion transport and homeostasis, protein localization, metabolic processes and ATP transport (Fig 2). Many of these proteins may reflect processes critical for sperm remodeling (e.g., transport processes) and these proteins would necessarily be removed in the final maturation processes occurring in the cauda epididymis.

**Table 5:** GO term enrichment in proteins added during epididymal transit^a^.

**Male gamete generation / Spermatid development & differentiation / Spermatogenesis**				
4930579C15Rik	Bsph1	Klhl10	Sept4	Tcp11
Acsbg2	Cabyr	Mycbp	Sgpl1	Theg
Adam1b	Catsperb	Nme5	Spata20	Tssk1
Akap14	Catsperg2	Pgam2	Spata6	Tssk6
B4galnt1	H2afx	Prkaca	Spink2	Txndc3
Bag6	Htt	Psme4	Sun5	Txndc8
Bax	Immp2l			
**Sperm capacitation**				
Bsph1	Cabyr	Catsperb	Prkaca	Sept4
**Sperm-egg & sperm-ZP Binding**				
Adam1b	B4galt1	Cct7	Spa17	Zp3r
Adam3	Cct3	Cct8	Spaca3	
**High-density lipoprotein particle clearance**				
Gpld1	Lipg			
**Mannose metabolic process**				
Man2a2	Man2b2	Mpi	Pmm2	Tsta3
Man2b1	Man2c1			
**Response to nitrosylative stress**				
Adh5	Gclm			
**Flagellar cell motility**				
Ccdc39	Ccdc40			
**Mitochondrial protein processing**				
Afg3l1	Immp2l			

^a^Cytoscape was used to identified enriched protein groups compared to proteins identified as "removed" (see Fig 2).

#### Segment-specific Proteins

**Fig 3:**
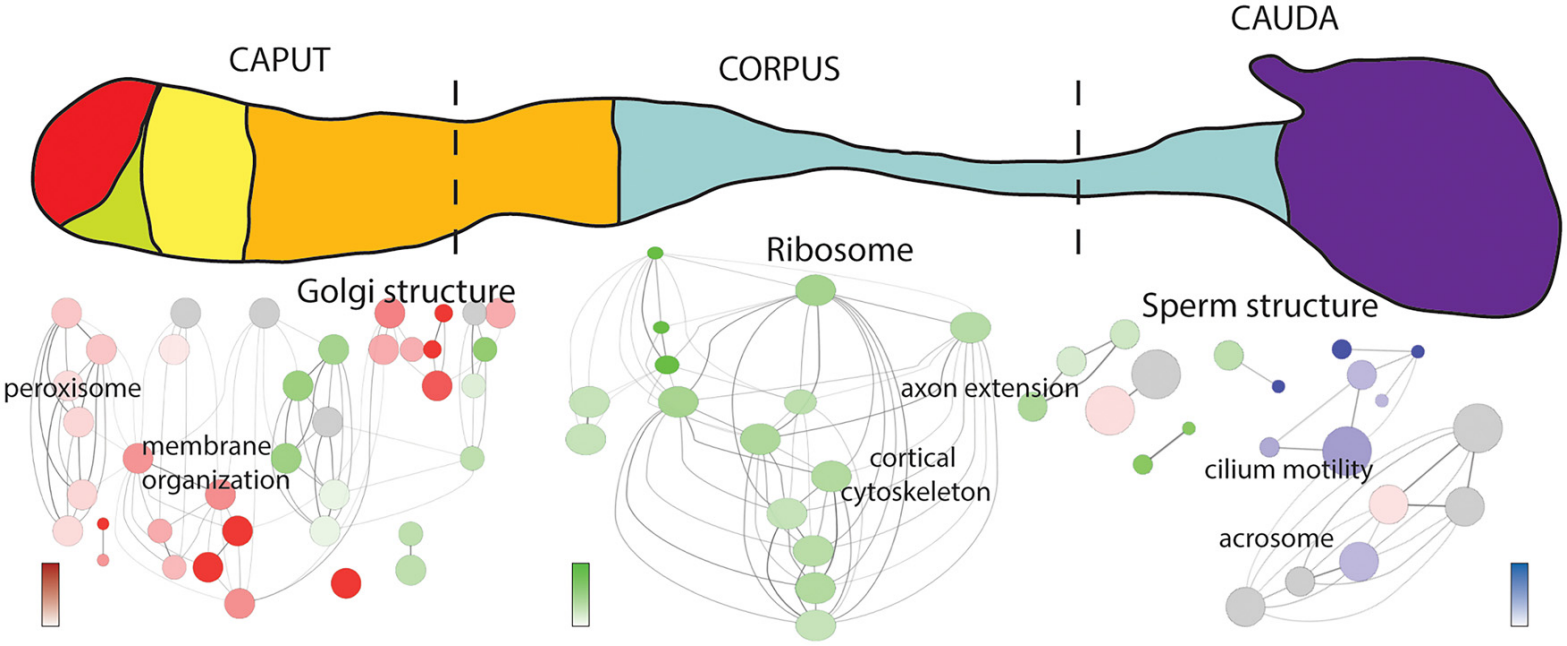
Region-specific enrichment of proteins unique to each segment and organized by GO categories related to cell location. Enriched categories are color-coded: caput (red), corpus (green) and corpus (blue) indicating the general overall differences in these protein datasets. The darker the node, the greater enrichment in the indicated categories. White, or gray-colored nodes indicate no specific enrichment between each of the three protein lists.

Sperm proteins specific to each of the three segments provide additional insights into the dynamics of sperm maturation as proteins unique to, or predominantly present in, specific regions of the epididymis may reflect regional-specific functions. Analysis of the patterns of protein additional and loss in the three sperm proteomes (Fig 1B) identified 296, 263 and 158 proteins uniquely identified in the caput, corpus and cauda sperm proteomes, respectively (S3 Table). To identify putative regional-specific protein functions inherent in these lists, a comparative network analysis was performed to determine enriched GO cell component categories between each list (Fig 3). A clear pattern of enrichment for unique GO categories was observed in each segment consistent with the overall changes in proteome composition (Fig 1).

#### Immune System Process / Immunity Genes

**Fig 4:**
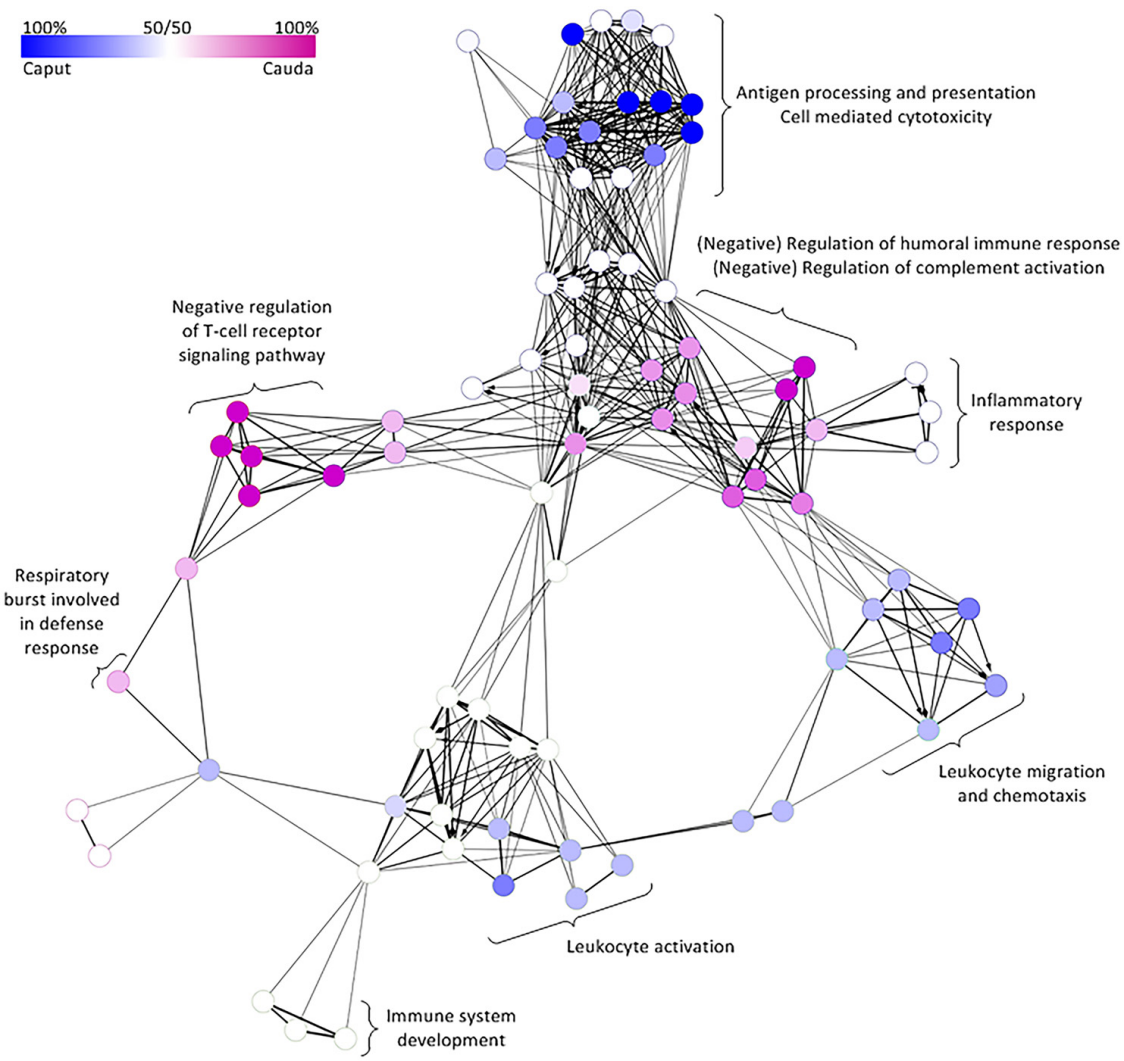
Enriched immune system process GO categories for proteins in the caput and cauda sperm proteomes. Network immune system process diagram depicting GO categories enriched in the caput (blue) and cauda (magenta) sperm proteomes. The darker the node color, the stronger the enrichment while white nodes indicate an equal abundance of that functional category in the list of added and removed proteins.

Previous proteomics studies have identified a variety of immunity proteins in sperm (reviewed in [42]). Using a network analysis, we investigated the distribution of immune system process categories in the caputSP, as compared to the caudaSP (Fig 4). We found that the caputSP and caudaSP are enriched for proteins involved in different immune system processes. For example, proteins involved in antigen processing and presentation, cell mediated cytotoxicity, leukocyte migration and chemotaxis, and leukocyte activation are enriched in the caputSP (blue nodes, Fig 4). Meanwhile, proteins involved in respiratory burst involved in defense response, negative regulation of T-cell receptor signaling pathways, regulation of humoral immune response and regulation of complement activation are enriched in the caudaSP (magenta nodes, Fig 3). Proteins involved in the inflammatory response, as well as immune system development are present in an equal proportion in both the caputSP and caudaSPs (white nodes, Fig 4).

During analysis of immunity proteins in the mouse caputSP, corpusSP and caudaSP it became apparent that β-defensins were not annotated as part of the GO Immune System Process ontology. β-defensins are antimicrobial peptides that play a role in activating both innate and adaptive immune responses but have also evolved important roles in fertility and many also exhibit testis specific expression. We identified 9 β-defensins in the caputSP, corpusSP and caudaSPs, including DEFB2, 11, 19, 20, 22, 23, 29, 47, and sperm associated antigen 11B (SPAG11B). Interestingly, β-defensin mRNA expression patterns in the epididymis correlate with the corresponding protein ID by MS (Table 6). For example, DEFB2 expression is maximal in the cauda epididymis precisely where the protein was identified, and DEFB23 expression is maximal in the corpus, also correlating with the MS data. DEFB20 expression was highest in the caput with measurable expression along the entire epididymis in concert with MS identification in all three sperm proteomes. Of the 9 β-defensins identified in this study, only DEFB22 and SPAG11b have characterized functions in reproductive biology.

**Table 6:** The β-defensins identified in the mouse sperm proteome.

β-defensin	Identified by MS in this Study			Highest Expression	Human Ortholog	Sperm Function(s)
	Caput SP	Corpus SP	Cauda SP			
Defb2			X	Cauda	-	Unknown
Defb11		X	X	Cauda	-	Unknown
Defb19		X	X	Caput / Corpus	-	Unknown
Defb20	X	X	X	Caput	Defb128^1^	Unknown
Defb22		X		Corpus	Defb126^2^	Penetration through cervical mucus, ZP binding, component of sperm glycocalyx
Defb23		X		Corpus	Defb129^1^	Unknown
Defb29		X		Corpus	Defb116	Unknown
Defb47	X			Unknown	Defb130^3^	Unknown
Spag11B	X			Unknown	Spag11A^4^, Spag11B^4^	Sperm motility and maturation

Segments of the epididymis and associated protein identified and regions of highest gene expression (based on transcriptional data from the mouse & rat) are shown. Known human orthologs and sperm functions are indicated.

^1^ 1:1 ortholog

^2^ 1:1 ortholog, apparent

^3^ 1:many ortholog

^4^ Possible ortholog

## Discussion

Shotgun proteomics has proven useful for discovery-based studies of the sperm proteome, and in the present study LC MS/MS was used to identify the broad based changes in protein functional categories during sperm maturation in the epididymis. The mouse epididymis is distinguished by three morphological regions, a proximal (caput) and two distal segments (corpus, cauda) as shown diagrammatically in Fig 1. Although convenient, use of these morphological landmarks for the dissections introduces some unavoidable variation in our analyses. The level of variation was minimized by the use of epididymides from 5 mice as our starting material. Given that our analysis was designed to provide an overall picture of the changes occurring in the sperm proteome during sperm maturation, the impact of variation should be minimal. Additionally we note that due to costs and equipment access constraints, the present study utilized MS datasets acquired from single replicate experiments for each of the pooled epididymal segments reported, and therefore limits our ability to provide accurate quantitative information on individual protein levels. However, because we were interested in acquiring a systems-level comparative view of the overall changes occurring in the epididymis during maturation, we employed strict criteria for peptide and protein identification that provided a solid, and conservative foundation for our comparative studies. Despite these limitations, our approach provides two advantages; first, the strict requirements used provided high confidence in assignments of proteins IDs to each segment, and second, the large-scale patterns of functional protein groupings identified in each segment provide compelling statistical evidence of the overall veracity of the results as discussed further below.

LC MS/MS identified 1536, 1720, and 1234 proteins in the caputSP, corpusSP, and caudaSP, respectively. Previous studies have characterized the mouse caputSP and caudaSP, however, no prior study has characterized the corpusSP or analyzed the proteome of sperm obtained from multiple epididymal segments in the same study. A previous study of the mouse caputSP identified 205 proteins, of which 54% (110 proteins) were also identified in the caputSP in this study [4]. In addition, a previous study [2] of the mouse caudaSP identified 858 proteins (808 after the removal of protein variants [4]), of which 57% (460 proteins) were identified in the caudaSP. A more recent study identified 2850 proteins in the mouse caudaSP [21] of which 883 proteins were identified in the caudaSP in this study, representing an overlap of 31%. Although it is noted that each of these studies employed different sample preparation techniques, methods for peptide and protein identification and mass spectrophotometers, the resulting overlap for the datasets was 31–57% (S4 Table).

Interestingly, in terms of the number of proteins, sperm proteome complexity rose from caput (1536 proteins) to a maximum in the corpus (1720) and then decreased in the cauda (1234). While the nature of these dynamic changes in sperm composition is yet to be determined, this study does establish the basic efficacy of using a high throughput proteomics approach to the study of sperm maturation in a mammalian model system. Given that the study examined three broad morphological regions, details of sperm protein acquisition and loss during maturation await more refined studies of sub-regions of the epididymis.

Across the epididymis, 765 proteins are common to all three segments defining a provisional ‘core’ sperm proteome from which proteins are added and removed during sperm maturation. Interestingly, more proteins were removed (1034) than added (732) during transit through the epididymis. Thus the proteomic evidence suggests the cellular mechanisms involved in the removal of sperm proteins represent an important and underappreciated component of the maturation process.

In total, 47 proteins were identified in the caputSP and caudaSP which were not identified in the corpusSP. This subset likely represents proteins which are present in the corpusSP but missed during MS analysis. Overall this number of proteins represents only 2–3% of the total number of proteins identified and only 4–5% of proteins which were identified as added or removed from sperm during epididymal transit in this study, providing an internal estimate of the false-positive rate and further supporting our overall conclusions. To our knowledge, this study is the first to employ a systems-level approach to identify proteins added and removed from sperm during epididymal transit and serves to further expand our knowledge base of proteomic dynamics during sperm maturation.

### Correlation with previous studies

Over the past few decades the biochemistry and cell biology of specific epididymal proteins has been studied (reviewed in [15]). However, the present study is one of the few systematic studies of sperm proteome maturation and represents the first such study of all three major segments of the epididymis. Although our study idenfied a substantial dataset of proteins and documented changes in the sperm proteome during the maturation process, we wished to validate the dataset by comparing our high-throughput analyses with previously described studies. To this end we utilized an online text mining tool Biocontext [43] that identified 102 literature references containing contextual information on proteins in the mammalian epididymis (S5 Table). Manual curation found 19 references that studied proteins found in the MmusSP. In all cases the published data positively correlated either directly or indirectly with the MmusSP dataset. For example, based on spectral counts, changes in levels of ADAM7, Crisp-1 and Crisp-2 in the MmusSP precisely correlated with prior studies of at both the expression and protein levels (S5 Table). Thus this limited level of analysis provides some validation of the dynamic changes observed in the sperm proteome during epididymal maturation.

### Epididymosmes

Epididymosomes, membranous vesicles secreted by the epididymal epithelium into the epididymal lumen, are thought to be a major mechanism through which proteins are added to sperm during epididymal transit is (reviewed in [13]). Several studies have focused on determining the proteomic composition of epididymosomes in different species including the rodent, equine and bovine systems [44–46]. We compared our epididymal sperm proteome protein lists with proteins identified in a study of epididymosome proteins collected from the bovine caput and cauda epididymal lumen [47]. Using a strict one-to-one homology criteria to identify potential orthologs matches, we identified four potentially interesting proteins, DEFB119, MAN2B1, TXNDC8, and PDCD6, common to both the bovine epididymosome and our mouse datasets. These proteins had complementary expression patterns, found only in the epididymosomes caput fractions and were present only in the corpus and cauda sperm proteome fractions. This suggests that these proteins may be added to sperm by epididymosomes located in the proximal epididymis, and that addition of these proteins to sperm during transit through the proximal epididymis may be conserved across distantly-related species. The low number of protein found in our search clearly underpins the need for addtional high throughput studies designed to identify protein dynamics in both epididymis and sperm fractions.

Another study on the protein composition of human epididymosomes collected from patients during vasectomy reversal identified 146 different proteins [48]. Many of the proteins that were identified in human epididymosomes were also identified in sperm from all three segments of the mouse epididymis, including ADAM7, AKAP3, GAPDHS, ENO1, LDHC, and TEKT3. ADAM7 has previously been identified in epididymosomes isolated from the mouse, and has been shown to be transferred to sperm *in vitro* [8]. However, it remains unclear whether such proteins are added to sperm by epididymosomes during epididymal transit, or whether epididymosomes play a role in increasing or decreasing the abundance of these proteins in sperm, and what role changes in protein abundance play in sperm maturation in the epididymis.

CRISP1 was identified in human epididymosomes [48] and was likewise identified in the mouse corpusSP and caudaSP but not the caputSP. CRISP1 is known to be secreted into epididymal fluid, and has also been shown to be associated with cauda, but not caput sperm in the rat [10]. Taken together, these data suggest that CRISP1 is acquired by sperm during epididymal transit, and that this sperm modification may be conserved between a number of species, including the mouse, rat, and human.

CD9 was identified as a component of human epididymosomes [48] and also identified in the mouse caputSP but not the corpusSP or caudaSP. This suggests that epididymosomes may play a role in removing CD9 from the sperm surface during epididymal transit.

Overall it is difficult to draw meaningful conclusions when comparing epididymosome composition with sperm proteomes from distantly related species such as human and bovine. However it does represent a first step in this direction and, to our knowledge, this current study represents the first such comparison between these datasets. Clearly, future directions aimed at a thorough description of the protein composition of mouse epididymosomes isolated from the caput, corpus, and cauda epdidymal lumen would complement and extend this study and greatly enhance our understanding of sperm maturation in the mouse.

### GO Analyses

A number of GO categories are differentially enriched in the caputSP, corpusSP and caudaSP. Compared to the caudaSP, both the caputSP and corpusSP are highly enriched in proteins involved in localization, including lipid and protein localization. Several studies have documented that the lipid composition of sperm plasma membranes changes during epididymal transit, which is thought to lead to the increase in membrane fluidity observed in mature sperm [49]. Consistent with this, several biological process terms related to lipid metabolism, including lipid biosynthetic process, lipid catabolic process, lipid modification, and lipid transport are highly enriched in the caputSP and corpusSP. These GO categories are significantly reduced in the caudaSP suggesting that the major elements of epididymal lipid reorganization in sperm occurs primarily in the caput and corpus epididymis, and they are subsequently degraded or lost during the final stages of the maturation process in the cauda epididymis.

Components of the translation machinery, including many structural components of the ribosome, were found enriched in the caputSP, corpusSP and caudaSP. However, the data indicates a gradient of enrichment in these GO terms from proximal to distal along the epididymis. Ribosomal components have also been reported in other sperm proteomic datasets [2,4,7,21], suggesting that sperm may possess some level of translation capacity within the epididymis. It has been demonstrated that mRNAs exist in post-testicular sperm, some of which are transferred to the egg at the time of fertilization [50]. Sperm-derived mRNAs may play a role in capacitation and in the early zygote development. It is thus possible that some mRNAs in sperm are translated during epididymal transit and contribute to the complex maturation process.

#### Proteasome Proteins

Components of the proteasome have been described in macaque, human and mouse sperm proteomes [4,7,12]. Consistent with these findings we identified proteasome proteins in all three segments of the epididymis including components of the 26S proteasome (consisting of the 20S core and 19S regulatory particle) and alternate proteasome activator PA200. Also identified were PSME1 & 2 components of an additional alternate proteasome activator, PA28, a known element of the immunoproteasome complex. The PA28 subunit enhances the ability of the 20S proteasome to produce peptides that can be bound by MHC class I molecules [51]. Identification of PSME1 & 2 in sperm is interesting because gametes are thought to lack MHC class I molecules on their surface, however previous studies have identified a number of proteins involved in MHC class I antigen presentation in sperm [42]. For example, β2 microglobulin (β2m), a component of the MHC class I receptor, was identified in the mouse sperm proteome [4]. Further, BAG6, a protein involved in antigen processing and presentation of antigens by the MHC class I receptor, was identified in this study and has previously been identified in the mouse sperm proteome [4]. To date, the function of these proteins in sperm has yet to be elucidated, and it remains unclear why these proteins are components of sperm in the absence of MHC class I presentation in this cell type.

#### Immune System Processes

Proteomics studies of sperm have identified a number of different immunity proteins, some of which exhibit sperm specific function, while the function of many others remains to be elucidated (reviewed in [42]). In this study the immunity proteins identified in the caputSP and caudaSP were enriched in different functional categories, with the caudaSP enriched in proteins involved in immune system processes related to the respiratory burst involved in defense responses, negative regulation of T-cell receptor signaling pathways, regulation of humoral immune response and regulation of complement activation. These processes may play important roles in sperm biology directly, or may facilitate the interaction of sperm with mucosal immune cells located in the female reproductive tract. For example, the respiratory burst reaction is typically orchestrated by phagocytic cells of the innate immune system. This pathway has also been implicated in sperm capacitation, however excessive generation of ROS in sperm has also been associated with infertility [52,53]. The other enriched immune system processes, including negative regulation of T-cell receptor signaling pathways, regulation of humoral immune response and regulation of complement activation likely have to do with the regulation of the immune response in the female reproductive tract by sperm. For example, sperm are known to be able to regulate the complement pathway that is highly active in the female reproductive tract (reviewed in [54]).

Although not functionally annotated in the GO Immune System process ontology, we identified a number of β-defensins in the caputSP, corpusSP and caudaSPs (Table 5). Defensins are antimicrobial peptides which can activate both the innate and adaptive immune response and play a role in a number of processes including development and fertility [55]. The β-defensin gene family has rapidly expanded in the mammalian lineage as a result of gene duplication, resulting in numerous species and lineage specific proteins that evolved an array of specialized functions [55]. Many β-defensins exhibit testis- or epididymis-specific expression and several studies have identified region-specific expression of β-defensins along the length of the epididymis in different species, including the mouse, rat and human [16,28,56].

In total, we identified 9 β-defensins in the caputSP, corpusSP and caudaSPs, including Defb2, 11, 19, 20, 22, 23, 29, 47, and sperm associated antigen 11B (SPAG11B), a gene that arose from the fusion of two ancestral β-defensin genes [57]. Interestingly, the region of the epididymis that exhibits the highest expression for each β-defensin gene correlates extremely well with the region in which we first identify the protein (Table 5). Of the 9 β-defensins identified in this study, only two have known sperm specific functions including DEFB22 and SPAG11B. DEFB126, the human ortholog of mouse DEFB22, is a widely studied β-defensin plays a role in sperm penetration through cervical mucus and ZP binding, and SPAG11B is involved in sperm motility and maturation [58–61]. The functions of the remaining β-defensins is unknown, however, since most of these proteins are specifically expressed in the epididymis they may play a role in the immune response to epididymal pathogens, or have sperm specific functions which have yet to be elucidated.

## Supporting Information

S1 Fig Spectral count profile of the top 20 proteins in the cauda. Each of the 20 most abundant cauda sperm proteins (green bars) were compared across each of the three epididymal segments. Note that 17 proteins were identified in all 3 segments and that, in general cauda sperm protein abundance was many fold greater than in the other 2 segments.
(TIF)

S1 Table The epididymal sperm proteome. All proteins identified in the caput, corpus and cauda sperm are listed with additional genomic, spectral count and coverage information. A listing of all 765 proteins common to all three segments is also listed.
(XLSX)

S2 Table Listing of added and removed proteins in each segment. Proteins added or removed during epididymal transit, inferred from the core sperm proteome (see Materials and Methods) for each segment. Also listed are the 47 proteins common to the caput and cauda but not found in the corpus.
(XLSX)

S3 Table Unique segment-specific sperm proteins. Listing of the 296, 263, 158 proteins uniquely identified in the caput-, corpus- and cauda-sperm proteomes, respectively.
(XLSX)

S4 Table Overlap with other published mouse sperm proteomes. (DOCX)

S5 Table Correlation of the MmusSP with previously described epididymal proteins. Literature examples demonstrating correlation between gene and/or protein expression with the sperm proteome in the epididymis.
(XLSX)
