# Supplementary figures and images for: Sperm Proteome Maturation in the Mouse Epididymis
Source: PLoS One. 2015 Nov 10;10(11):e0140650. doi: 10.1371/journal.pone.0140650 (PMC4640836; doi:10.1371/journal.pone.0140650)

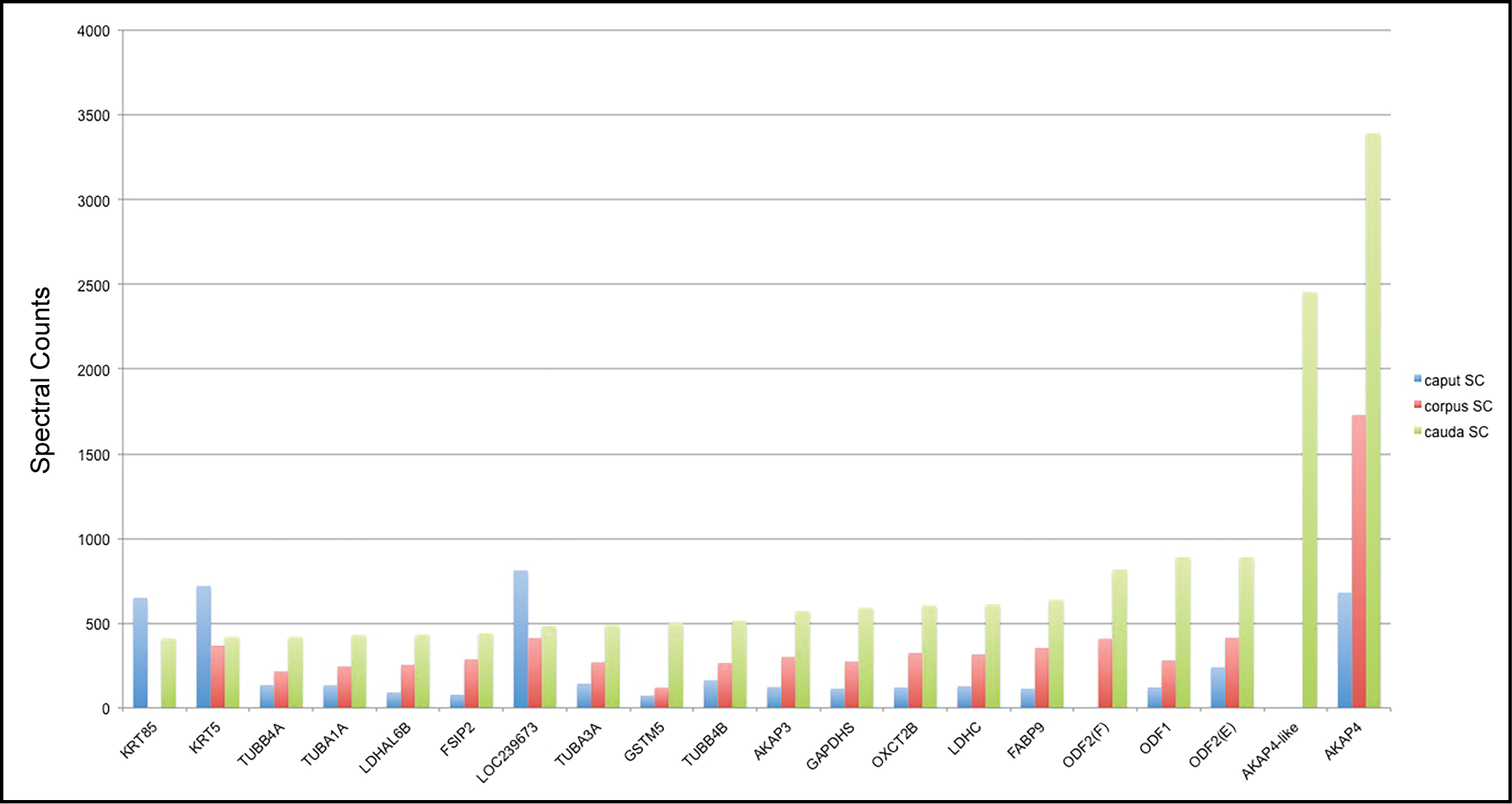

Supplement: S1 Fig — Each of the 20 most abundant cauda sperm proteins (green bars) were compared across each of the three epididymal segments. Note that 17 proteins were identified in all 3 segments and that, in general cauda sperm protein abundance was many fold greater than in the other 2 segments. (TIF) [file pone.0140650.s001.tif]
