# Supplementary material for: Sperm Proteome Maturation in the Mouse Epididymis
Source: PLoS One. 2015 Nov 10;10(11):e0140650. doi: 10.1371/journal.pone.0140650 (PMC4640836; doi:10.1371/journal.pone.0140650)
Supplement: S4 Table — (DOCX) [file pone.0140650.s005.docx]

**Supplemental Table 4. Overlap with other published sperm proteomes**

|  | **CAPUT SP** | **CORPUS SP** | **CAUDA SP** |
| --- | --- | --- | --- |
| **Current Study** | 1642 | 1817 | 1345 |
|  | | | |
| **OVERLAP** (# proteins identified in study/overlap with current study/% overlap) | | | |
| **Dorus et al. 2010** | 205 / 117 / 57% | - | - |
| **Chauvin et al. 2012** | - | - | 2850 / 965 / 72% |
| **Baker et al. 2008** | - | - | 858 (808*) / 472 / 58% |

Overlap of proteins identified in the caput, corpus and cauda SP in the current study with mouse sperm proteomes identified in previous studies. Table includes whole cell proteomes only, and does not include studies of sperm subcellular components. No previous studies of the corpus SP have been reported.
